# Exploring cannabinoid receptor CB1 autophagy and the obesity phenotype of p62-deficient mice

**DOI:** 10.1016/j.bbrep.2026.102571

**Published:** 2026-04-02

**Authors:** Christina Keller, Sebastian Rading, Bahar Candur, Laura Bindila, Gabriele Loers, Meliha Karsak

**Affiliations:** a RG Neuronal and Cellular Signal Transduction, Institute of Human Genetics, University Medical Center Hamburg-Eppendorf, Martinistr. 52, Hamburg, 20246, Germany; b Clinical Lipidomics Unit, Institute of Physiological Chemistry, University Medical Center of the Johannes Gutenberg University Mainz, Mainz, 55128, Germany

**Keywords:** Endocannabinoid system, CB1 receptor, p62, Obesity, Autophagy, Locomotor activity, Fasting

## Abstract

The endocannabinoid system (ECS) and the autophagy receptor p62 are both implicated in metabolic regulation and obesity, yet the mechanisms linking these pathways remain unclear. Here, we investigated whether p62 modulates CB1 receptor (CB1R) turnover or function and whether CB1R contributes to the metabolic phenotype of p62 knockout (KO) mice. In primary cortical neurons from wild-type mice, inhibition of autophagic flux with Bafilomycin A1 led to substantial CB1R accumulation, demonstrating that CB1R is a subject to autophagy-dependent degradation. CB1R agonist stimulation partially reduced this accumulation, suggesting receptor activation influences turnover. In vivo, p62 deficiency did not significantly alter CB1R protein abundance in the brain or hypothalamus, although hypothalamic ERK1/2 signaling downstream of CB1R was modestly attenuated. P62 KO mice displayed late-onset obesity without hyperphagia, early hypoactivity, and elevated hypothalamic 2-arachidonoylglycerol (2-AG) levels with age. Fasting–refeeding experiments revealed reduced food intake in adult and aged, but not juvenile, p62 KO animals. Pharmacological CB1R antagonism did not uncover a direct receptor-dependent mechanism underlying these phenotypes. Together, these findings indicate that, although CB1R undergoes autophagic degradation in neurons, p62 deficiency does not alter steady-state receptor levels and does not directly account for obesity or hypoactivity in p62 KO mice. Within the scope of the experiments performed, CB1R is therefore unlikely to be a primary driver of the metabolic phenotype associated with p62 deficiency.

## Highlights

• Neuronal CB1R turnover depends on autophagy.
• p62 KO reduces refeeding with age.
• 2-AG is elevated in hypothalamus.
• Early hypoactivity precedes obesity.

## 1 Introduction

Obesity is a multifaceted disorder characterized by excessive fat accumulation, leading to various health complications such as insulin resistance, type 2 diabetes, and cardiovascular disease [1]. In recent years, significant attention has been given to the molecular pathways involved in the development and progression of obesity, with a particular focus on signaling mechanisms that regulate energy homeostasis. Among the critical players involved in these processes are the endocannabinoid system (ECS) and the autophagy machinery, which are increasingly recognized for their role in modulating metabolic pathways and cellular turnover [2,3].

The ECS comprises cannabinoid receptors (CB1R and CB2R), endogenous lipid ligands (endocannabinoids), and enzymes responsible for their synthesis and degradation. It plays a vital role in regulating numerous physiological processes, including appetite, energy metabolism, and lipid storage [[4], [5], [6]]. The cannabinoid receptor 1 (CB1R), a G-protein-coupled receptor (GPCR) particularly expressed on the presynaptic side of excitatory and inhibitory neurons in the cortex, hippocampus, hypothalamus, cerebellum and basal ganglia, is the most widely studied receptor of the ECS due to its prominent role in controlling food intake and body weight regulation [7,8]. Upon activation by its endogenous ligands - primarily 2-arachidonoylglycerol (2-AG) and anandamide (N-arachidonoylethanolamide or AEA) or by other agonists - CB1R stimulates various signaling cascades that promote hyperphagia, energy expenditure, and lipid accumulation [7,[9], [10], [11], [12]]. Additionally, activation of CB1R leads to short-term depression and long-term depression of synaptic transmission and activation of CB1R at the presynaptic terminal decreases transmission during neuronal activity by inhibiting synaptic vesicle release [13]. Dysregulation of CB1R signaling has been closely linked to the development of obesity and metabolic syndrome [14] as well as to neurological disorders [15].

Pharmacological modulation of CB1R has been explored as a potential therapeutic strategy to combat obesity. One notable example is Rimonabant (SR141716A), a selective CB1R antagonist that was developed to inhibit CB1R activity, thereby reducing food intake and body weight [16,17]. Clinical trials with Rimonabant showed promising results in weight reduction, improved metabolic profiles, and reduced abdominal fat [16]. However, due to adverse psychiatric effects, including depression and anxiety, the drug was eventually withdrawn from the market [18]. Nonetheless, these studies underscored the central role of CB1R in metabolic regulation and its potential as a therapeutic target for obesity and associated metabolic disorders.

The hypothalamus plays a central role in regulating food intake, energy expenditure, and body weight through complex neuroendocrine pathways. Within the hypothalamus, CB1R signaling exerts potent effects on appetite regulation and energy homeostasis by modulating the activity of key neurotransmitter systems [12]. Activation of CB1R in the hypothalamus is known to increase food intake, while CB1R antagonism suppresses appetite [5]. Additionally, CB1R signaling has been implicated in the regulation of physical activity, with CB1R activation leading to a reduction in locomotor behavior [19,20]. Of note, CB1R availability in homeostatic and reward-related brain regions like the hypothalamus, cortex and midbrain is negatively correlated with the body mass index [21] and obesity leads to alterations in gray matter volume in cortical regions involved in reward processing and inhibitory control, taste processing and interoception, aversive states, and learning/memory [[22], [23], [24], [25], [26]].

While CB1R signaling has been well characterized in the context of appetite regulation and energy expenditure, less is known about the mechanisms governing its turnover and degradation. Given that CB1R is a GPCR, its activity is tightly regulated by mechanisms such as receptor desensitization, internalization, and lysosomal degradation [27,28]. The turnover of CB1R is thought to involve several pathways, including ubiquitin-proteasome-mediated degradation and autophagy [29]. Autophagy, a catabolic process responsible for the degradation of damaged proteins and organelles, is essential for maintaining cellular homeostasis. The effects of cannabinoid receptors on autophagy have been extensively investigated [30]; however, the converse, whether and how the receptors themselves undergo autophagic processing, including transport and degradation within autophagosomes remains less understood.

The autophagy machinery relies on specialized proteins, including p62 (also known as sequestosome 1), which serves as an autophagy receptor responsible for identifying and targeting specific cargo for degradation. p62 plays a crucial role in linking ubiquitinated proteins to the autophagosomal-lysosomal degradation pathway [[31], [32], [33]]. Several studies have revealed that p62 deficiency leads to the development of obesity, insulin resistance, and leptin intolerance in mice, establishing a critical role for p62 in the regulation of energy balance [34,35]. Specifically, p62 knockout (KO) mice exhibit an age-dependent obesity phenotype, with increased body weight, fat accumulation, and liver steatosis as they age [35,36]. Despite in-vivo studies highlighting age-dependent obesity, the underlying cellular mechanisms which are essential for targeted drug development remain unknown.

Based on our previous work identifying p62 as a protein–protein interaction partner of peripheral CB2R using protein precipitation and mass spectrometry [37], and considering the established roles of the autophagy receptor p62 and of the ECS in energy regulation, we investigated whether CB1R undergoes autophagy-dependent turnover and whether p62 contributes to the regulation of CB1R function in the central nervous system. Specifically, we addressed whether CB1R degradation is linked to autophagy, whether CB1R signaling is altered in p62-deficient mice, and whether such changes are associated with the development of obesity.

To this end, we combined in vitro analyses of CB1R turnover in primary neurons with in vivo studies of CB1R signaling, endocannabinoid levels, locomotor activity, and feeding behavior in p62 knockout mice. We show that CB1R undergoes autophagy-dependent turnover in neurons, and that p62 deficiency is associated with altered endocannabinoid signaling, reduced locomotor activity, and the development of obesity independent of hyperphagia.

## 2 Results

### 2.1 Autophagy and CB1R protein turnover in neurons

Given the established role of p62 in autophagy, we hypothesized that p62 might regulate CB1R expression levels and degradation via the autophagosomal-lysosomal pathway, thereby potentially influencing the development of age-dependent obesity in p62 KO animals. We therefore first asked whether CB1R protein turnover is linked to autophagic flux in primary neurons.

**Fig. 1:**
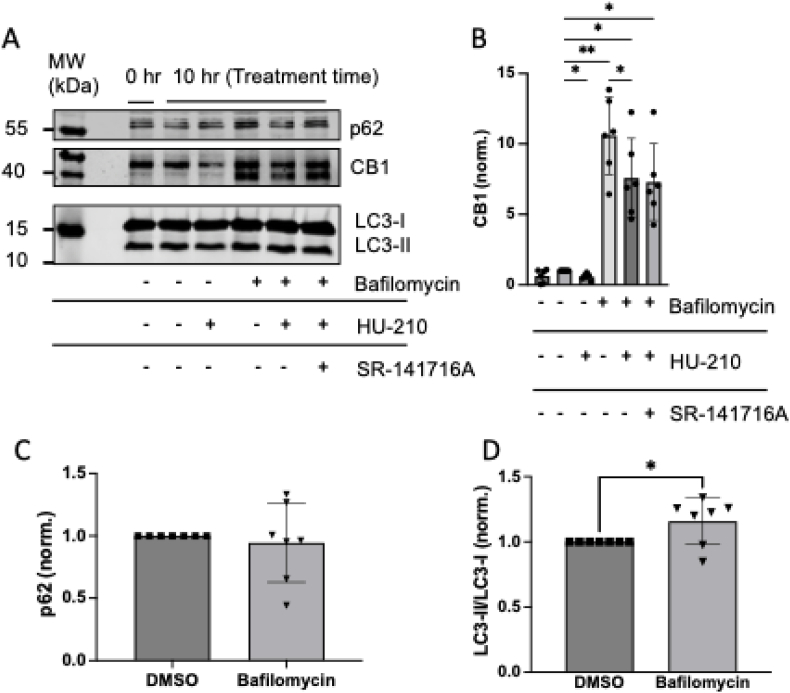
CB1R protein turnover is autophagy mediated. Dissociated cortical neurons from mouse embryos at embryonic day 15.5-16.5 were studied to investigate CB1R autophagy. The cells were stimulated with CB1R agonist HU-210 (300 nM) and antagonist SR-141716A (300 nM) in combination with Bafilomycin A1 (100 nM), an inhibitor of autophagosome-lysosome fusion. Western blot signals of CB1R (A) were normalized to total protein amount (LICORbioTM total stain) and quantified using Empiria Software (B-D) and revealed a significant increase in CB1R levels (B), no change in p62 levels (C) and an increase in the LC3-II/LC3-I ratio (D) after inhibition of autophagic flux by 10 h treatment with Bafilomycin A1. One-way repeated measurement ANOVA (one-way ANOVA) and Dunnet's multiple comparison testing were applied to make comparisons between non-treated and Bafilomycin A1 and HU-210 or SR-141716A treated cortical neurons (B) and t-test was applied for comparisons of DMSO and Bafilomycin-treated samples (C + D) (one variable; ∗p < 0.05, ∗∗p < 0.01). N = 6 independent embryonic cortical cultures and independent experiments were used in this study. A representative Western blot is shown.

Bafilomycin A1 treatment of cultured wild-type cortical neurons resulted in a significant accumulation of CB1R protein, with levels increasing up to 10-fold, indicating that CB1R undergoes active degradation through autophagy (Fig. 1A and B). HU-210 CB1R agonist stimulation reduced the Bafilomycin-induced accumulation of CB1R, suggesting that CB1R activation triggers desensitization mechanisms that counteract receptor accumulation, even when autophagic degradation is inhibited. The CB1R antagonist SR141716A did not block the agonist effect efficiently (p-value = 0.24). Following treatment with Bafilomycin A1 no change in p62 (Fig. 1A–C) but an increase in LC3-II (Fig. 1A–D) protein level was observed confirming that autophagy was blocked. The appearance of the second lower band of CB1R (Fig. 1A) might derive from immature proteins or degradation products [38].

Our findings demonstrate that CB1R proteins undergo turnover and that autophagic degradation plays a key role in the flux of CB1R proteins in primary neurons, suggesting that receptors that mediate cargo delivery to the autophagosome may facilitate this process. p62 is an ideal candidate for this role, given its well-established involvement in autophagy and cargo transport. However, p62 dependence was not directly tested in vitro, representing a limitation of the study.

Dissociated cortical neurons from mouse embryos at embryonic day 15.5-16.5 were studied to investigate CB1R autophagy. The cells were stimulated with CB1R agonist HU-210 (300 nM) and antagonist SR-141716A (300 nM) in combination with Bafilomycin A1 (100 nM), an inhibitor of autophagosome-lysosome fusion. Western blot signals of CB1R (A) were normalized to total protein amount (LICORbio^TM^ total stain) and quantified using Empiria Software (B-D) and revealed a significant increase in CB1R levels (B), no change in p62 levels (C) and an increase in the LC3-II/LC3-I ratio (D) after inhibition of autophagic flux by 10 h treatment with Bafilomycin A1. One-way repeated measurement ANOVA (one-way ANOVA) and Dunnet's multiple comparison testing were applied to make comparisons between non-treated and Bafilomycin A1 and HU-210 or SR-141716A treated cortical neurons (B) and *t*-test was applied for comparisons of DMSO and Bafilomycin-treated samples (C + D) (one variable; ∗p < 0.05, ∗∗p < 0.01). N = 6 independent embryonic cortical cultures and independent experiments were used in this study. A representative Western blot is shown.

### 2.2 p62 KO mice develop obesity without hyperphagia

Due to our previous findings of a direct protein-protein interaction between the peripheral cannabinoid receptor CB2R and p62 ^37^, we wanted to investigate whether CB1R expression is altered in p62 KO mouse brain, assuming that disturbances to CB1R flux might affect CB1R protein levels. We hypothesized that the deletion of p62 could lead to modulated CB1R flux, resulting in enhanced CB1R expression in p62 KO tissue. We therefore first assessed body weight and food intake during the period of obesity onset.

**Fig. 2:**
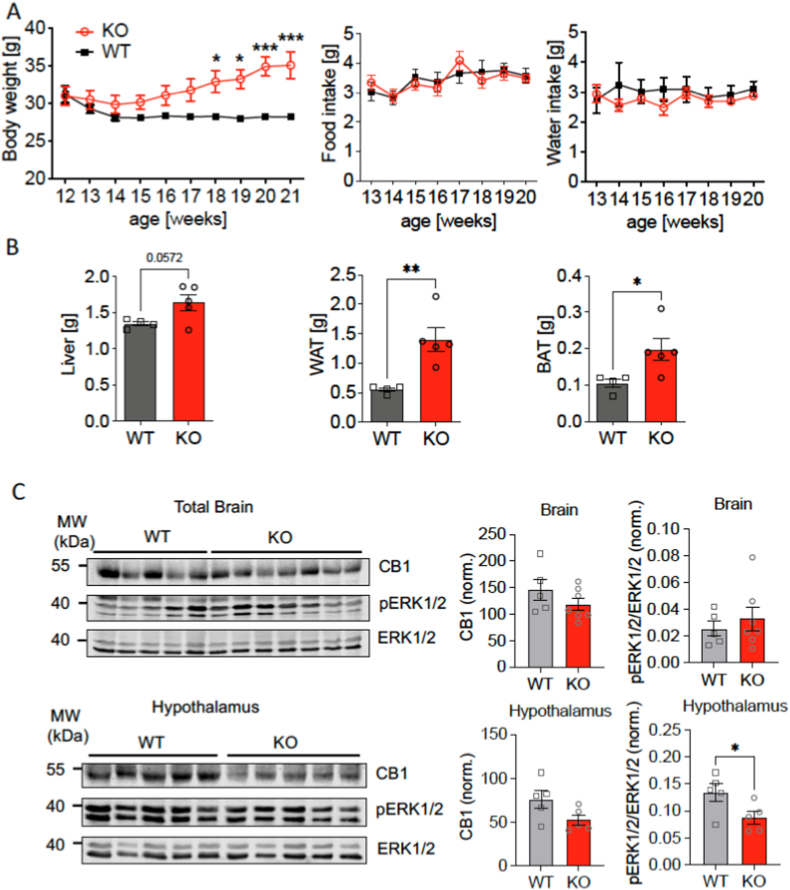
Lack of hyperphagia during obesity onset in p62 KO mice and normal CB1R protein levels. Body weight, food and water intake, liver, WT and BAT weight were determined in male p62 KO mice and WT male littermates. (A) Body weight of p62 KO mice increased more strongly between 12 and 21 weeks of age compared to WT animals. Daily food and water intake of male p62 KO mice was comparable to that of WT mice. (B) Total liver weight showed a tendency to be higher and adipose tissue (white = WAT and brown = BAT) was significantly increased in 21-week-old male p62 KO mice compared to their WT littermates. (C) Western Blot detection of CB1R, ERK1/2 and pERK 1/2 in whole brain and hypothalamic protein lysates of 7-months old male p62 KO and WT mice. Quantification of levels of CB1R normalized to total protein revealed no difference between genotypes in tissue lysates while levels of pErk1/2 were lower in the p62 KO hypothalamus. Data was analyzed by unpaired t-test (B, C) and by using 2-way ANOVA with repeated measures and Bonferroni adjusted p-values, ∗p < 0.05, ∗∗p < 0.01, ∗∗∗p < 0.001 (A). All error bars show mean ± SEM. In (A, B) WT male N = 4; KO male N = 5 and in (C) WT N = 5; KO male N = 5. MW = molecular weight marker.

As observed before, male WT and p62 KO mice had a similar body weight by the start of the observation at 12 weeks of age (Fig. 2A; WT, 31,2 g ± 1,1g; KO, 31,0 g ± 1,3 g). As mice aged it became apparent that p62 KO mice gained more weight compared to their age matched WT littermates which was significant by 18 weeks of age (Fig. 2A; WT, 28.3 g ± 0.1 g; KO, 32.9 g ± 1 g). The weight of p62 KO mice further increased to a difference in weight of more than 6 g between p62 KO and WT mice by the end of observation period at 21 weeks of age (Fig. 2A). In addition, daily food intake increased with age in both genotypes; however, p62 KO mice showed no signs of hyperphagia during obesity onset since they ate a similar amount of food as their WT littermates (Fig. 2A). WT and p62 KO mice consumed a similar amount of about 3 ml of water daily. Water consumption was almost unaltered during the whole testing period for both genotypes (Fig. 2A). The impact of obesity on organs of p62 KO mice was significant at 5 months of age as p62 KO mice showed increased organ weight. The weight difference of freshly removed liver was nearly significant between p62 KO and WT mice (Fig. 2B). However, the amount of total freshly removed WAT and BAT was significantly increased in p62 KO mice compared to WT littermates (Fig. 2B).

Taken together, no hyperphagia was detectable in p62 KO mice during obesity onset and therefore it cannot be the cause of the obesity phenotype.

### 2.3 CB1R abundance is unchanged in brain and hypothalamus of p62 KO mice

To investigate whether altered CB1R expression or signaling contributes to the phenotype of p62 KO mice, we performed Western Blot analyses to assess CB1R protein levels in the total brain and hypothalamus of p62 KO and WT mice (Fig. 2C).

Our analysis revealed comparable CB1R levels in whole-brain lysates between p62 KO and WT mice (Fig. 2C, upper panel). Similarly, no significant differences in the levels of phosphorylated ERK1/2 in the CB1R downstream signaling pathway were detectable in total brain lysates. In hypothalamic lysates CB1R levels were slightly lower in male p62 KO mice, but this difference did not reach statistical significance (Fig. 2C lower panel: WT: 76 norm ±23 norm, n = 5; KO: 53 norm ±13 norm, n = 5; p = 0.08). However, ERK1/2 activation by phosphorylation was significantly reduced in the hypothalamus of p62 KO mice (Fig. 2C lower panel: WT: 0.13 ± 0.05, n = 5; KO: 0.09 ± 0.02, n = 5; p = 0.05), while total ERK1/2 levels were comparable.

These findings suggest that the absence of p62 does not directly impact overall CB1R protein expression or expression in the hypothalamus.

### 2.4 Reduced locomotor activity precedes obesity in p62 KO mice

**Fig. 3:**
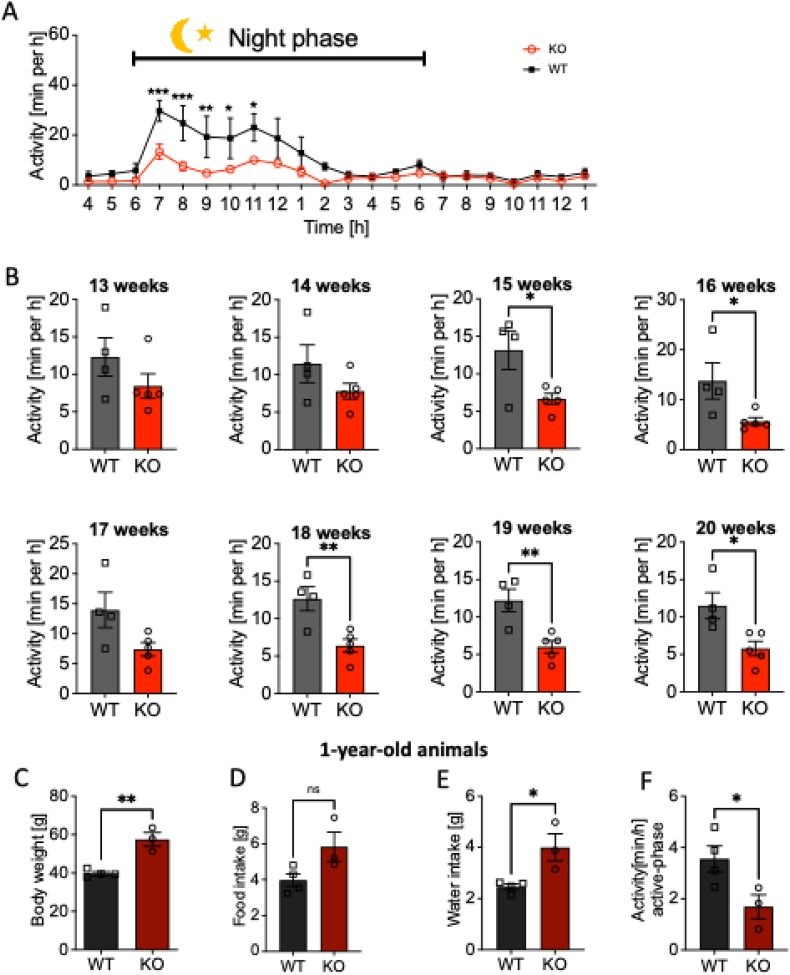
P62 KO mice show reduced home cage activity during their active phase before onset of obesity. (A) Active and inactive phase of male p62 KO and WT mice at an age of 16 weeks. Mice were more active during the night (red-illumination 6 p.m. to 6 a.m.) and less active during the day (bright light 6 a.m. to 6 p.m.) as expected. P62 KO mice were less active during night compared to their WT littermates. In the following graphs only the active phase (6 p.m. to 6 a.m.) was analyzed and compared between male p62 KO and WT mice. (B) Voluntary locomotion of p62 KO mice was significantly reduced beginning at 15 weeks of age. WT male N = 4; KO male N = 5. (C) Body weight was significantly increased in one-year-old male p62 KO mice compared to their WT littermates. (D) Food intake of one-year-old male p62 KO mice was mildly but not significantly increased compared to WT mice. (E) Water intake of p62 KO mice was significantly increased in p62 KO mice. (F) Home cage activity during the active phase (red illumination 6 p.m. to 6 a.m.) showed reduced activity of p62 KO compared to WT mice. 1-year-old male WT and p62 KO mice were observed for 5 days and the average activity was analyzed. WT male N = 4; KO male N = 3. Data was analyzed by unpaired *t*-test (B–F) and using 2-way ANOVA with repeated measures and Bonferroni adjusted p-values in (A) (∗p < 0.05, ∗∗p < 0.01, ∗∗∗p < 0.001). All error bars show mean ± SEM.

Since CB1R stimulation influences locomotion [39], and it is well-documented that older p62 KO animals exhibit reduced locomotion (as described by Ref. [40]), we sought to investigate whether locomotion is already reduced in young p62 KO animals and how this reduction develops as they age (Fig. 3). We hypothesized that reduced locomotor activity might underlie the obesity observed in p62 KO mice.

Locomotor activity was assessed throughout the critical developmental period (weeks 13 to 20), during which obesity emerges. Initially, no significant differences were observed between the two groups (Fig. 3A and B). However, by around 15 weeks of age (see Fig. 3B), three weeks before significant weight gain began, p62 KO mice showed a notable decrease in voluntary activity. This decline persisted throughout the study period, suggesting that reduced physical activity is an early event in the development of obesity in these mice. Notably, the reduction was most pronounced during the dark phase, when activity levels are typically highest. The consistently lower activity profile of p62 KO mice compared to their WT littermates reinforces the idea that diminished locomotion is a contributor to their excessive weight gain.

To further assess this hypoactive phenotype, we examined voluntary locomotion in 1-year-old male p62 KO (N = 3) and WT (N = 4) mice. At this age, p62 KO mice displayed a pronounced obesity phenotype, weighing 18 g more than their WT littermates (Fig. 3C). Despite their increased body weight, p62 KO mice showed only a modest increase in food intake (+1.8 g, p = 0.07; Fig. 3D) and water consumption (+1.5 g, p = 0.02; Fig. 3E) relative to WT controls. Interestingly, both groups consumed approximately 10% of their body weight in food, indicating that hyperphagia alone does not account for the obesity observed in p62 KO mice. A significant decrease in voluntary locomotion was observed in 1-year-old male p62 KO mice during their active phase (Fig. 3F). On average, p62 KO mice were active for just 1.7 min per hour, whereas WT mice were active for almost twice as long (3.6 min per hour). Although both genotypes exhibited a decline in locomotor activity with age, the hypoactive phenotype in p62 KO mice was apparent before severe obesity developed, becoming even more pronounced by 1 year of age. These findings confirm that obesity in p62 KO mice is not driven by hyperphagia, but is instead closely linked to reduced locomotor activity.

### 2.5 Hypothalamic 2-AG levels are increased in p62 KO mice

Next, we investigated the levels of endocannabinoids, as they play a crucial role in modulating CB1R function. Since CB1R activation is largely dependent on its endogenous ligands, examining the endocannabinoid levels in various tissues was essential for understanding whether altered signaling contributed to the observed phenotypes in p62 KO mice. Specifically, we focused on the hypothalamus, liver, and WAT, as these regions are key regulators of energy balance, food intake, and metabolism.

**Fig. 4:**
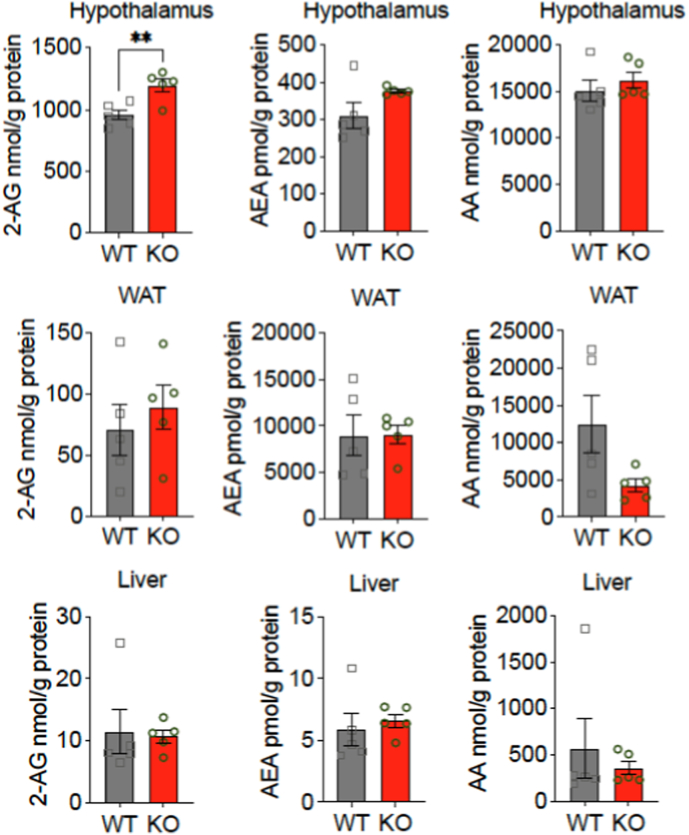
Increased 2-AG levels in the hypothalamus of p62 KO mice. Endocannabinoid 2-AG was significantly increased in the hypothalamus of p62 KO mice, while anandamide (AEA) and arachidonic acid (AA) were comparable between genotypes. Endocannabinoid measurement showed no significant difference for 2-AG, AEA and AA in WAT of WT and p62 KO mice. Data was analyzed by unpaired *t*-test, ∗ ∗∗p < 0.01. All error bars show mean ± SEM. All mice were male and aged 7 months. WT male N = 5; KO male N = 5.

In the hypothalamus, we observed a significant increase in 2-AG levels in male p62 KO mice compared to WT controls (Fig. 4). Interestingly, AEA and AA levels remained unchanged between genotypes (Fig. 4). In WAT, 2-AG and AEA concentrations were comparable between p62 KO and WT mice, while AA levels showed a mild but non-significant decrease in p62 KO mice. Similarly, endocannabinoid levels in liver tissue did not differ between genotypes. However, 2-AG levels were elevated in the hypothalamus of p62 KO mice. In contrast, levels of AEA and AA remained unchanged across all examined tissues (Fig. 4). As CB1R activation is known to suppress locomotion and promote lipid storage, these elevated hypothalamic 2-AG levels could explain both the decreased activity and increased adiposity observed in p62 KO mice. The unchanged endocannabinoid levels in WAT suggest that the metabolic effects of p62 deficiency are likely to be mediated through central rather than peripheral CB1R pathways.

### 2.6 Reduced fasting-induced food intake in p62 KO mice despite intact CB1R signaling

Our data strongly suggest that the reduced physical activity observed in p62 KO mice precedes the onset of obesity, rather than being a consequence of increased body weight. Given CB1R's role in regulating food intake following fasting, we were prompted to investigate whether p62 KO animals exhibit a modulated response to food intake after an 18-h fasting period.

**Fig. 5:**
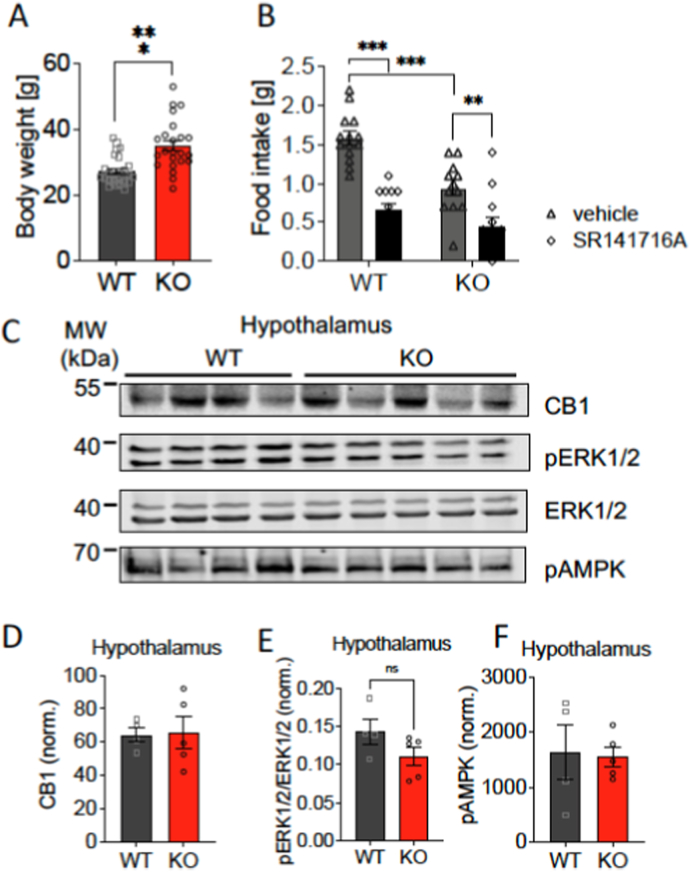
P62 KO mice show reduced food intake after 18 h of fasting. Comparison of body weight, food intake and protein levels of female p62KO mice and their female WT littermates. (A) Body weight was increased in p62 KO females compared to WT females. (B) Mice were fasted for 18 h and the amount of food eaten during the 3 h of food access was measured. Mice treated with SR141716A (i.p.) ate less in comparison to vehicle treated mice. However, feeding was reduced in p62 KO mice (vehicle) compared to control WT mice (vehicle). 5 to 9 months old females were used in this experiment (WT N = 25; KO N = 23) and were further divided in control and treatment group (WT vehicle N = 14, WT SR141716A N = 11; KO vehicle N = 12, KO SR141716A N = 11). (C, D) An additional group aged 7 months was sacrificed directly after 18 h of fasting and Western Blot quantification of CB1R was performed. CB1R levels, normalized to total protein levels, were not altered between female p62 KO and WT mice (WT N = 4, KO N = 5). (E) In addition, ERK1/2 activation in the hypothalamus was evaluated by Western Blot and showed similar ERK1/2 activation in p62 KO and WT mice after fasting. (F) Analysis of the hypothalamus of p62 KO and WT mice after fasting showed comparable AMPK phosphorylation in the Western Blot. Data was analyzed by using unpaired t-test (A and D) and 2-way ANOVA with Bonferroni adjusted p-values, ∗p < 0.05, ∗∗p < 0.01, ∗∗∗p < 0.001 (B). All error bars show mean ± SEM. MW = molecular weight marker.

First, we investigated whether female mice show a similar phenotype as the p62 KO males, and as anticipated, the p62 KO female mice (N = 23) weighed significantly more than female WT mice (N = 25) (+7 g, p < 0.001; 5–9 months old females; Fig. 5A). In this experiment, female mice were allowed access to food pellets during their “breakfast time,” followed by an 18-h food deprivation period. Afterwards, they were administered either vehicle or the CB1R antagonist SR141716A (3 mg/kg *i.p.*) and given controlled food access for the next 3 h during the subsequent “breakfast time”.

As expected, female WT mice treated with SR141716A consumed significantly less food than vehicle-treated WT controls (Fig. 5B). Interestingly, control p62 KO mice consumed significantly less food than their WT counterparts. Moreover, food intake in female p62 KO mice treated with SR141716A was further reduced, confirming that CB1R blockade was effective in this genotype.

To investigate whether CB1R signaling is altered in female p62 KO mice, we analyzed CB1R protein levels in hypothalamic lysates from a separate group of fasted (18 h) mice (7-month-old; WT N = 4, KO N = 5; Fig. 5C). No significant differences in CB1R levels were observed between the genotypes (Fig. 5D). Similarly, fasting-induced ERK1/2 and AMPK activation in the hypothalamus did not differ between WT and p62 KO mice (p = 0.15 and p = 0.88, respectively; Fig. 5E and F).

Interestingly, we observed that vehicle-treated female p62 KO mice already exhibited a reduction in food intake following the fasting period, a phenotype reminiscent of CB1R inhibition or deletion [41]. This led us to question whether young p62 KO animals, which do not yet exhibit significant body weight gain, would show the same response.

### 2.7 Reduced fasting-induced food intake in p62 KO mice emerges with age

**Fig. 6:**
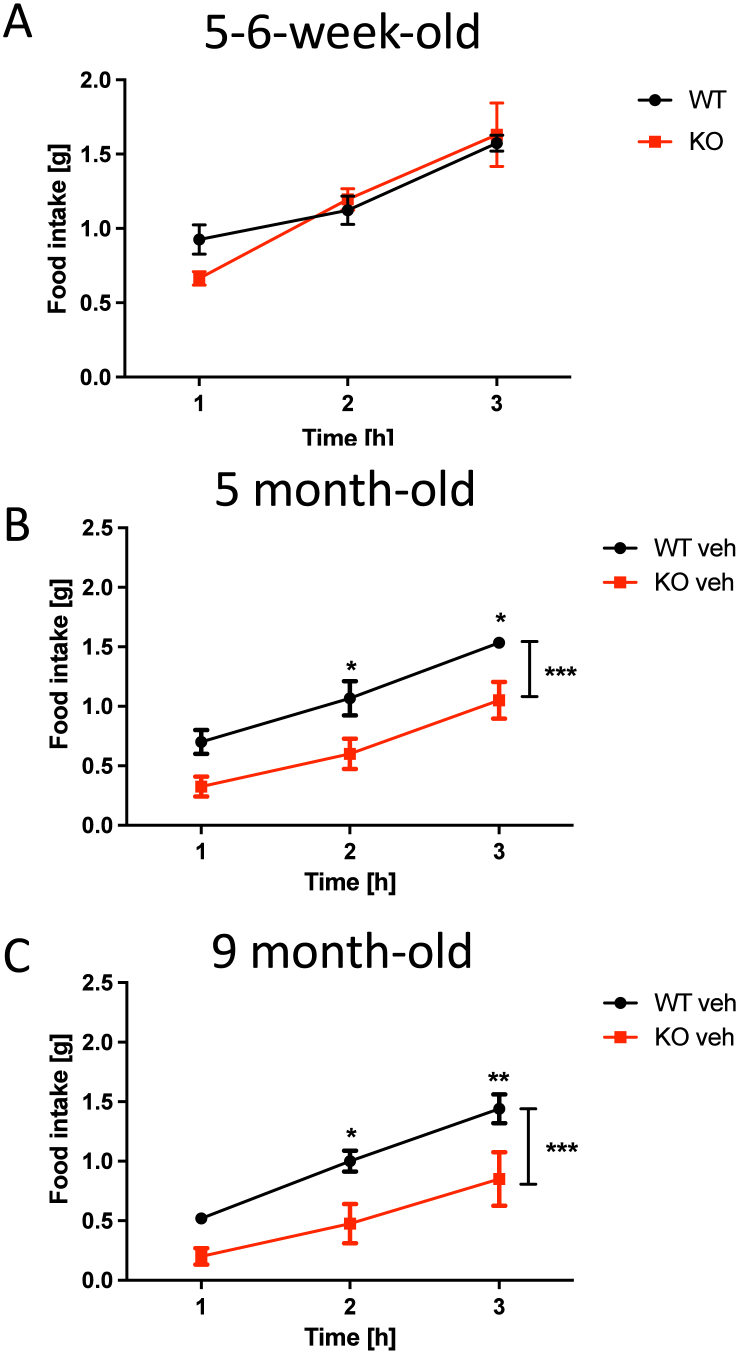
Age-dependent reduction of voluntary food intake after 18 h fasting in p62 KO mice. (A) Food intake after an 18-h fasting period was determined for the following 3 h in different age groups. At the age of 5-6 weeks p62 KO female animals (N = 5) ate the same amount as their WT controls (N = 7). (B) The food consumption for animals aged 5 month (WT N = 3, KO N = 4) and (C) aged 9 months (WT N = 5, KO N = 4) was reduced in female p62 KO mice. Data was analyzed by using 2-way ANOVA with Bonferroni adjusted p-values, ∗p < 0.05, ∗∗p < 0.01, ∗∗∗p < 0.001. All error bars show mean ± SEM.

In order to investigate fasting-mediated feeding, we examined how young female mice (aged 5–6 weeks) responded to a fasting challenge, comparing their food intake with that of the control groups in the CB1R antagonist experiment (Fig. 6A–C). The groups were separated into three age categories: juvenile (5–6 weeks old; Fig. 6A), adult (5 months old; Fig. 6B) and aged (9 months old; Fig. 6C). Unlike adult and aged p62 KO mice, juvenile p62 KO mice (5–6 weeks old) consumed similar amounts of food after the fasting period as their WT littermates.

Our findings suggest that adult and aged female p62 KO mice exhibit reduced food intake following a fasting challenge, and that p62 KO mice maintain responsiveness to CB1R antagonism.

## 3 Discussion

The present study was designed to address whether altered CB1R turnover or signaling contributes to the metabolic phenotype observed in p62-deficient mice given the established role of p62 in autophagy and prior evidence of its interaction with peripheral CB2R [37].

Our data demonstrate that neuronal CB1R is degraded via the autophagy-lysosome pathway, consistent with broader evidence that GPCRs can be substrates of autophagic degradation [42]. Previous work has primarily emphasized endocytosis and proteasomal degradation as the principal mechanisms for CB1R turnover [43], whereas the contribution of autophagy remains less explored for CB1R. Furthermore, previous studies investigated intensively the opposite effect namely the effect of CB1R on autophagy [30]. The robust accumulation of CB1R upon Bafilomycin A1 treatment in cultured neurons suggests that autophagy contributes significantly to CB1R homeostasis in neurons. In addition, the observed lower molecular weight CB1R band observed in immunoblots may represent immature or partially degraded receptor forms [38], further underscoring the dynamic flux of CB1R proteins within the degradative system.

The effect of HU-210 in reducing CB1R accumulation under Bafilomycin treatment suggests that receptor activation can modulate its autophagic fate. One possible explanation is that agonist-induced desensitization promotes recycling rather than degradation under autophagy-blocked conditions. Alternatively, agonist stimulation may engage alternative degradative pathways, such as endosomal degradation, partially compensating for impaired autophagy [44]. The weak antagonistic effect of SR141716A indicates that CB1R internalization and desensitization involve complex regulatory networks not blocked by inverse agonism. Furthermore, the high (10-fold increased) levels of CB1R, together with its appearance in different maturation/degradation states, may explain why it cannot be blocked by 300 nM of the receptor antagonist. These findings highlight the need for more detailed analyses of receptor trafficking pathways, ideally using live-cell imaging with fluorescently tagged CB1R, to distinguish recycling, proteasomal degradation, and autophagy-dependent turnover.

Although p62 is a canonical adaptor protein linking polyubiquitinated cargo to autophagosomes via LC3 interaction, our experiments did not reveal significant differences in overall CB1R protein levels between p62 KO and WT mice. This result suggests that p62 may not be strictly required for CB1R degradation, at least at steady-state conditions. Another possibility is that compensatory mechanisms operate in p62 KO mice [34]. Other selective autophagy receptors such as NBR1 (neighbor of BRCA1 gene 1) could partially substitute for p62 [45] in mediating CB1R turnover, preventing overt accumulation of CB1R protein. Our biochemical analyses were limited to total CB1R protein levels conducted on bulk tissue lysates and did not distinguish between surface, internalized, and autophagosomal receptor pools. Furthermore, cell-type–specific effects of p62 deficiency could have been masked in our experiments, thus single-cell or neuron-type–specific approaches would help to clarify whether CB1R turnover is selectively altered in distinct neuronal populations. Additionally, co-immunoprecipitation or proximity ligation assays could directly test for CB1R-p62 interactions in the brain, extending our previous finding of a CB2R-p62 interaction [37]. Although CB1R protein levels were unchanged, hypothalamic ERK1/2 activation downstream of CB1R signaling was reduced in p62 KO mice.

Consistent with previous reports [35,46], we confirmed that p62 KO mice develop late-onset obesity. Importantly, we observed that this phenotype was not accompanied by hyperphagia or excessive water intake, indicating that energy imbalance is unlikely to result from increased caloric intake. Instead, reduced locomotor activity emerged as an early and sustained feature of p62 KO mice, preceding significant weight gain. This observation aligns with the findings of Müller et al. [40], who reported decreased movement in adipocyte-specific p62 KO mice, and underscores the role of p62 in regulating energy expenditure.

Interestingly, the hypoactive phenotype of male p62 KO mice resembles the known effects of CB1R activation, which suppresses locomotion and promotes energy storage [5,19,20]. This similarity suggests a potential functional link between p62 deficiency and enhanced CB1R signaling. Although our biochemical analyses did not detect an increase in CB1R, we found elevated hypothalamic 2-AG levels in p62 KO mice, which could drive hyperactivation of CB1R independently of receptor abundance. Elevated 2-AG may explain both the reduced locomotion and the enhanced adiposity of p62 KO mice, highlighting the importance of the endocannabinoid tone as a mediator between p62 deficiency and CB1R physiology. While an increased endocannabinoid tone could theoretically influence locomotion and metabolism, the absence of receptor upregulation and the preserved antagonist responsiveness argue against a primary CB1R-driven mechanism.

Our fasting-refeeding experiments revealed that adult and aged female p62 KO mice consumed less food following fasting compared to WT controls. This reduction was further potentiated by CB1R antagonism, indicating that CB1R signaling remained functional in the absence of p62. Notably, juvenile p62 KO mice did not exhibit altered feeding responses, suggesting that the effect is age-dependent. One possible explanation is that compensatory mechanisms preserve normal CB1R function in young animals but deteriorate with age, leading to progressively altered feeding behavior. In support of this possibility, previous studies have reported reduced hypothalamic CB1R expression in middle-aged WT mice compared to younger animals [47], as well as an age-related decline in CB1R coupling to ^35^S-GTPγS during aging [48]. However, whether similar age-dependent alterations in CB1R expression, coupling efficiency, or downstream signaling occur in p62 KO mice remains unknown. This should be systematically investigated in future studies, including direct assessments of receptor abundance on single cell level, G-protein coupling, and signaling efficacy across age groups.

## 4 Conclusion

Collectively, our data indicate that although CB1R is an autophagy substrate in neurons, p62 deficiency does not substantially alter CB1R abundance or core receptor responsiveness in vivo. Therefore, CB1R does not directly account for the metabolic phenotype observed in p62 KO mice under the experimental conditions investigated here.

## 5 Materials and methods

### 5.1 Experimental animals

Knockout-first (KO) p62 mice (C57BL/6N-Sqstm1^tm1a(KOMP)Wtsi^), sourced from the KOMP directory (ID: 41073; UC Davis, University of California, USA), were on a C57BL/6 N background (ID: 41073) and contain a promoter-driven selection cassette (lacZ and neomycin). These mice were crossed with C57BL/6J mice (Charles River) for more than six generations to produce fertile offspring with normal growth [49,50]. Mice were maintained at the animal facility of the Universitätsklinikum Hamburg-Eppendorf under a 12-h light/dark cycle, with a room temperature of 22 °C and 55% humidity. They were housed either individually with visual and olfactory contact to the other mice or in groups of up to five mice per cage in standard Makrolon type II cages (22 × 16 × 14 cm), which were equipped with bedding, nesting material, and provided with ad libitum access to standard food and water unless otherwise stated (e.g. during fasting experiments). Mice were fed with Altromin standard diet 1320 (# 1324; Altromin Spezialfutter GmbH & Co. KG, Lage, Germany) containing carbohydrates 2091 kcal/kg (65%), protein 768 kcal/kg (24%) and fat 367 kcal/kg (11%). All animal procedures were conducted by a blinded experimenter and in compliance with German and European regulations for the protection of experimental animals (“Principles of laboratory animal care”, NIH publication No. 86-23, revised 1985) and were approved by the Behörde für Gesundheit und Verbraucherschutz of the City of Hamburg (project identification code numbers 139_15, approved 17.01.2016; 154_16, approved 14.07.2016; and ORG_1022, approval date 31.07.2020).

### 5.2 Activity measurement

To assess their spontaneous home cage activity, male mice were individually housed for the duration of the experiments in standard Makrolon type II cages with minimal bedding and nesting material to facilitate observation using the Mouse-E-Motion infrared observation system (Infra-e-motion, Henstedt-Ulzburg, Germany), positioned above the cage. This system detected movement by measuring displacements of at least 1.5 cm from the initial position every second. Activity was recorded as minutes per hour. The system also measured water intake, while food intake was assessed by weighing the food pellets provided in the cage. Activity measurements were conducted over three consecutive days and repeated weekly with mice from 11 to 23 weeks of age. The average activity over the three days was used for analysis. Body weight was recorded weekly before initiating the activity experiments.

### 5.3 Fasting experiment

Female p62 KO and WT littermates at the age of 5 to 9 months (WT = 25, KO = 23) were individually housed, fasted for 18 h and afterwards received a intra peritoneal *(i.p.)* injection of either vehicle or SR141716A (3 mg/kg) using a 1 ml syringe and a G27 cannula. The weight of two food pellets was measured and the food pellets were placed into the cage of each mouse. The amount of food eaten was determined after 1, 2 and 3 h following the injection. SR141716A was dissolved in 0.2% Tween® 20 and DMSO, then diluted in sterile 0.9% NaCl solution for injection. The vehicle solution contained the same components, but without ligand. Another group of female mice aged 5-6 weeks (WT = 7, KO = 5) was fasted for 18 h and food consumption during the following 3 h was determined without any injections. Afterwards mice were sacrificed immediately, and organs were harvested.

### 5.4 Organ harvesting

Mice were anesthetized using a gas mixture of 80% CO_2_ and 20% O_2_, followed by 100% CO_2_ to ensure euthanasia. Reflexes were checked to confirm death, and the mouse was sprayed with 70% ethanol to prevent contamination. All organs, including visceral fat pads and skin, were carefully excised. Brain tissue, liver, WAT, and BAT were weighed immediately, frozen in liquid nitrogen, and stored at −80 °C for further analysis. Cortices were harvested from wild-type mouse embryos of either sex at E15.5-16.5. Cortical pieces were collected and further processed as described below.

### 5.5 Preparation of buffers/reagents and isolation of cortical neurons

Cortical neurons were chosen as model neurons for the in vitro studies. Cortical neurons are characterized by their relatively high levels of p62 protein [46], express considerable amounts of CB1R [51], contain similar neuron types as the hypothalamus and can be isolated with a high yield, which makes them suitable model neurons to investigate the p62-CB1R interplay.

The Neurobasal medium (Thermo Fisher Scientific, #21103049), 2 mM l-glutamine (Thermo Fisher Scientific, #25030081), 100 U/mL penicillin, and 100 μg/ml streptomycin, and 1% B27 supplement (Thermo Fisher Scientific, #17504044) were utilized for neuronal culture. For digestion, a solution of 0.5% trypsin (Sigma, #T9935-100 MG) in Hank's balanced salt solution (HBSS; company) was prepared, filtered, and stored at −20 °C. The stopping solution was composed of 1% bovine serum albumin (BSA) (Sigma, #A3059-100g) and 1% trypsin inhibitor (Sigma, #T-6522) in HBSS, filtered, and stored at −20 °C. For the precoating of the tissue culture plates a solution of poly-l-lysine (PLL) was prepared by dissolving the PLL powder (Sigma, #P6282-5 MG) in ultra-pure water to a final volume of 50 ml. To enhance cellular adhesion, each well of a 24-well plate was incubated at 4 °C for 16–18 h with 250 μl of the PLL solution, then washed with HBSS and utilized directly for the culture experiments.

Cortical neurons were isolated and maintained as described (e.g. Ref. [52]) and cortices from three E15.5-16.5 wild-type mouse embryos of either sex were pooled for each experiment. In brief, cerebral cortices were cut into several pieces, and incubated in HBSS containing 0.025% trypsin at 37 °C for 30 min. After centrifugation for 1 min at 1500 g and 4 °C, the supernatant was removed and to stop the enzymatic reaction, tissue pieces were incubated with 2 ml stopping solution for 1 min. After washing in HBSS, the tissue pieces were mechanically dissociated in culture medium using fire polished Pasteur pipettes. Dissociated cells were seeded at a density of 1 × 10^6^ cells/ml into PLL-coated 24-wells (2.5 × 10^5^ cells/well) and maintained at 37 °C and 5% CO_2_.

The compounds were solved in dimethyl sulfoxide (DMSO) yielding stock solutions of 50 mM HU-210 (Tocris #112830-95-2), 50 mM SR141716 (Tocris #0923), and 10 mM Bafilomycin A1 (Sigma #88899-55-2). The maximal DMSO concentration in the experiment was 0.3%. Control solutions were prepared accordingly with DMSO (maximal 0.3%). The cells were cultured overnight, after which they were treated with 100 nM Bafilomycin A1, 300 nM HU-210, and 300 nM SR-141716A, or a DMSO solvent control solution, respectively. Following a 10-h incubation period in duplicate wells, total protein was harvested and proteins from six independent culture preparations/experiments were used for subsequent protein analysis as described below.

### 5.6 Preparation of protein samples

*For the preparation of protein samples from neurons*, cells were resuspended in phosphate-buffered saline (PBS), with 500 μl PBS added to each 24-well. Following centrifugation, the supernatant was discarded, and the cell pellet was resuspended in lysis buffer containing 0.2% dodecyl maltoside (DDM) and phosphatase inhibitors as described previously [53]. After incubation at 4 °C, samples underwent a second centrifugation at 16,200×*g* for 15 min at 4 °C, and the supernatant was collected for Western blot analysis.

*For the preparation of protein samples from organs,* frozen tissue samples (−80 °C) were placed in Eppendorf tubes with 500 μl of 0.2% DDM lysis buffer and kept on ice. The samples were homogenized using a TissueLyser LT (Qiagen) at 4 °C with a shaking speed of 50 rpm for 4 min, followed by a 30-min incubation on ice. The homogenate was centrifuged at 16,200×*g* for 15 min at 4 °C. The supernatant was transferred to a new tube and stored at −80 °C until further analysis.

### 5.7 Quantification of protein concentration

Protein concentration was quantified using a BCA protein assay kit (Thermo Scientific, Pierce™ BCA Protein Assay Kit) following the manufacturer's protocol. Absorbance at 562 nm was measured using a μQuant microplate reader (BioTek Instruments, Inc., Vermont, USA).

### 5.8 Polyacrylamide gel electrophoresis and Western Blotting

For SDS-PAGE, 12% polyacrylamide gels were prepared and either used immediately or stored at 4 °C in a sealed plastic bag wrapped in wet tissue for up to 7 days. Protein lysates were diluted in 0.2% DDM lysis buffer to a concentration of 20-200 μg per sample, kept on ice, mixed with 4 μl of 6x Laemmli buffer (75 mM Tris-HCl/Tris Base, 9% SDS, 50% Glycerol, 9% mercaptoethanol, 0.075% Bromophenol blue), and vortexed. Protein ladder (4 μl) and samples (20 μl) were loaded into the wells. Gels were run at 80 V for 10 min, then at 110 V until the dye front reached the end of the gel. After electrophoresis, proteins were transferred onto nitrocellulose membranes at 95 V and 4 °C for 2 h. Membranes were air-dried overnight, rehydrated in Tris-buffered saline solution (TBS; 150 mM NaCl, 10 mM Tris) for 5 min, and rinsed in ddH_2_O. Membranes were stained with Revert™ 700 Total Protein Stain solution (LICORbio™) for 5 min, then washed with Revert™ 700 Wash Solution (LICORbio™). After rinsing in ddH_2_O, membranes were imaged at 700 nm using the Odyssey® CLx imaging system (LICORbio™), destained with 5 ml Revert™ Destaining Solution (LICORbio™), and blocked in 5% skim milk powder in TBS for 1 h at room temperature.

Primary antibody solutions were applied and membranes incubated overnight at 4 °C. Following washes in TBST (150 mM NaCl, 10 mM Tris and 0.025% Tween® 20 in dH_2_O), membranes were incubated with IRDye® 680LT- or IRDye® 800CW-coupled secondary antibodies (LICORbio™, 1:1000) for 1 h at room temperature. Membranes were washed again in TBST and imaged with the Odyssey® CLx imaging system (LICORbio™). Band intensities were quantified using EMPIRIA Studio Software (Version: 3.0.0.173, LICORbio™). The following primary antibodies were used: polyclonal anti-CB1 rabbit (1:100 in TBST) (Immunogenes), anti-p62 (1:1000 in TBST + 1% milk powder) guinea pig (Progen #GP62-C) and anti-LC3B rabbit (1:1000 in TBS containing 5% milk powder) (Sigma Aldrich #L7543). The CB1R antibody was described and validated [54].

It is important to note that we loaded equal volumes of protein extracts onto the gels, rather than equal amounts of protein. Consequently, apparent differences in overall lane intensity may reflect variations in protein concentration between extracts, rather than genuine differences in CB1R abundance. Normalization to the Revert total protein signal corrects for this variability, ensuring accurate quantification of target protein levels.

### 5.9 Endocannabinoid measurement

Endocannabinoid levels in brain samples of 7 months old male mice were measured following previously published protocols [49,55,56]. Briefly, internal standards (50 μl) and a solvent mixture of ethyl acetate and n-hexane (250 μl, 9:1, v/v) were added along with 300 μl 0.1 % formic acid to tissue samples and vortexed. The samples were then homogenized in a tissue lyzer (Qiagen) for 2 cycles at 30 Hz for 30 s. For adipose tissue and liver samples 4 μl DMSO and 20 μl isopropanol were additionally added to the samples before homogenization to ensure better solubility of high fat content. The samples were then centrifuged (2 × 15 min at 5000×*g*, 4 °C) and stored at −20 °C for 10 min. The upper organic phase was collected and transferred to 96-well plates (Eppendorf, Merck, Darmstadt). After solvent evaporation, the residue was reconstituted in 50 μl acetonitrile/water (1:1, v/v) for Liquid Chromatography (LC)/Multiple Reaction Monitoring (MRM) analysis.

For LC, 20 μl of the reconstituted extract was injected onto a Phenomenex Luna 2.5 μm C18(2)-HST column (100 × 2 mm) with a pre-column C18, 4 × 2 mm (Restek). The mobile phase consisted of acetonitrile with 0.1% formic acid, which was increased from 55% to 90% over 2 min, then maintained at 90% for 5.5 min.

Endocannabinoids were analyzed using a 5500 QTrap triple-quadrupole linear ion trap mass spectrometer (AB SCIEX) equipped with a Turbo V Ion Source. The analysis was conducted in ‘positive-negative-switching’ mode, monitoring the following MRM transitions: AEA, *m/z* 348.3 to 62.3; AEA-d4, *m/z* 352.3 to *m/z* 62.1; 2-AG, *m/z* 379.1 to *m/z* 287.2, and 2-AG d5, *m/z* 384.2 to *m/z* 287.2. Calibration solutions were prepared with high-purity standards and spiked with deuterated endocannabinoids. Quantification was performed using Analyst 1.6.1 software (ABSciex), and endocannabinoid concentrations were normalized to protein amount. Protein amount per sample was determined from BCA measurements of the aqueous phase.

### 5.10 Statistics

To analyze data, statistical comparisons between WT and p62 KO mice were performed using a two-tailed unpaired *t*-test. Normality was tested by built-in analysis of GraphPad Prism Software. Statistical significance was defined as ∗p < 0.05, ∗∗p < 0.01, ∗∗∗p < 0.001. For repeated measures over time, a two-way repeated measures ANOVA (2-way ANOVA) was applied. If this analysis revealed significant effects of genotype, interaction, treatment, or time, Bonferroni's multiple comparison post hoc test was used to determine specific group differences (∗p < 0.05, ∗∗p < 0.01, ∗∗∗p < 0.001). All statistical analyses and graphing for were performed using GraphPad Prism version 7 (GraphPad Software, California, USA). For the analysis of cortical neuron cell data, one-way repeated measures ANOVA was used, followed by Dunnett's multiple comparison test to compare non-treated cells with those treated with bafilomycin A1, HU-210, and SR-141716A using GraphPad Prism version 10 (GraphPad Software, California, USA). Data are presented as mean ± SD, with 'n' indicating the number of independent experiments or number of mice in each experiment.

## 6 Text writing

To improve the quality of our manuscript ChatGPT by Open AI and DeepL Write was used to polish the text. After using this tool, the authors reviewed and edited the content as needed and take full responsibility for the content of the publication.

## Funding

This work was supported by intramural funding. For eCBs analysis, the core facility unit of the Clinical Lipdiomcis Unit has partially funded the analysis and data processing.

## CRediT authorship contribution statement

**Christina Keller:** Conceptualization, Data curation, Formal analysis, Investigation, Methodology, Validation, Visualization, Writing – original draft. **Sebastian Rading:** Investigation. **Bahar Candur:** Data curation, Formal analysis, Investigation, Methodology, Visualization, Writing – review & editing. **Laura Bindila:** Data curation, Formal analysis, Investigation, Methodology, Validation, Writing – review & editing. **Gabriele Loers:** Data curation, Investigation, Methodology, Writing – review & editing. **Meliha Karsak:** Conceptualization, Formal analysis, Project administration, Supervision, Validation, Visualization, Writing – original draft.

## Declaration of competing interest

The authors declare that they have no known competing financial interests or personal relationships that could have appeared to influence the work reported in this paper.

## Data Availability

Data will be made available on request.
