# Supplementary figures and images for: Exploring cannabinoid receptor CB1 autophagy and the obesity phenotype of p62-deficient mice
Source: Biochem Biophys Rep. 2026 Apr 2;46:102571. doi: 10.1016/j.bbrep.2026.102571 (PMC13087580; doi:10.1016/j.bbrep.2026.102571)

Fig. 1A

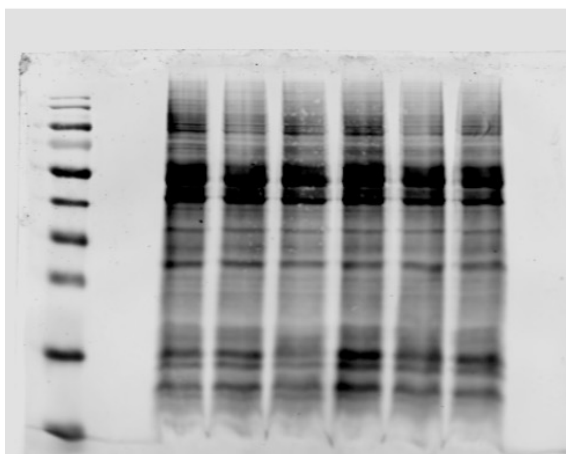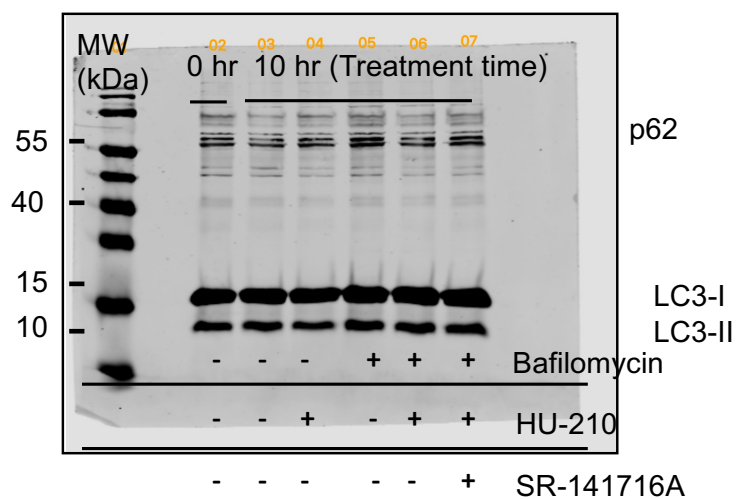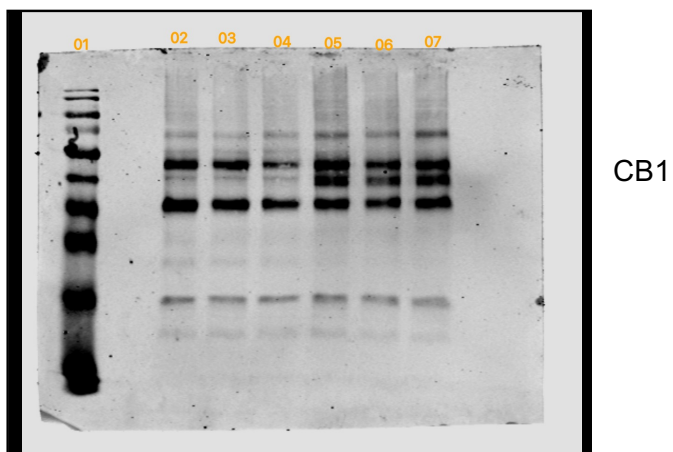

Fig. 2C upper

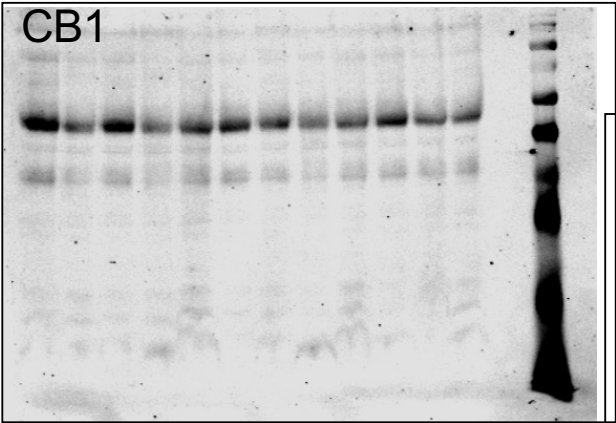

Total Brain  
WT      KO

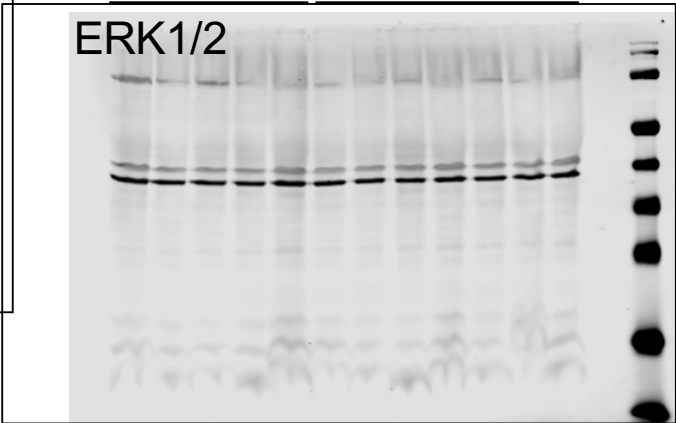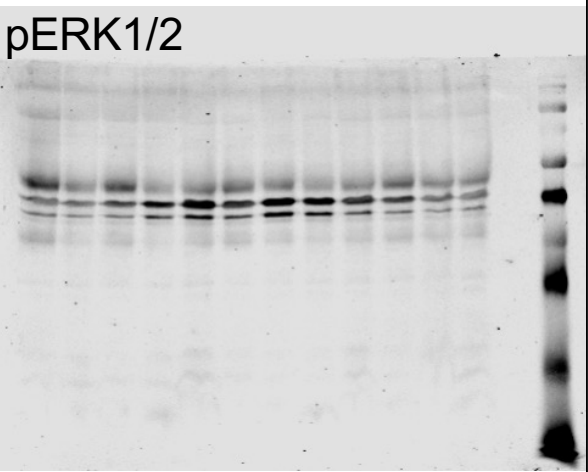

total

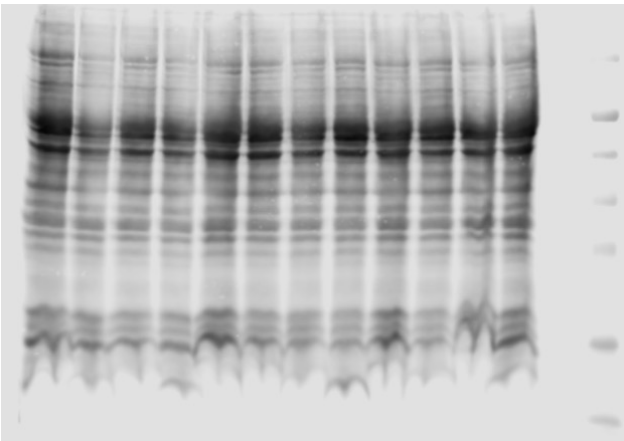

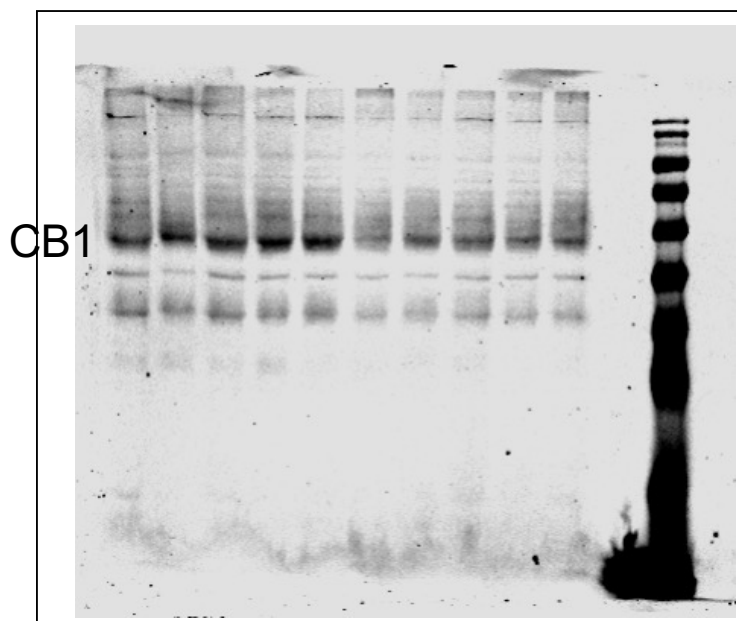

Fig. 2C lower / hypothalamus  
total

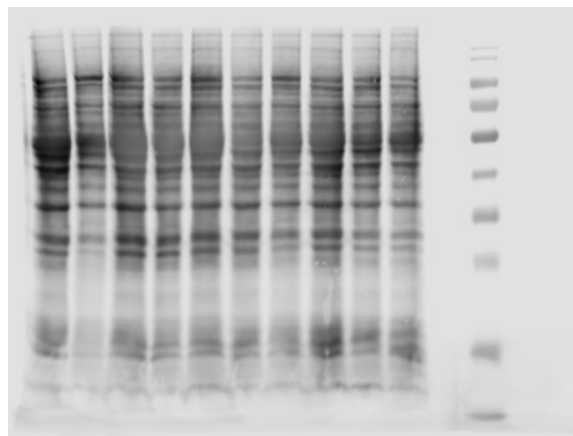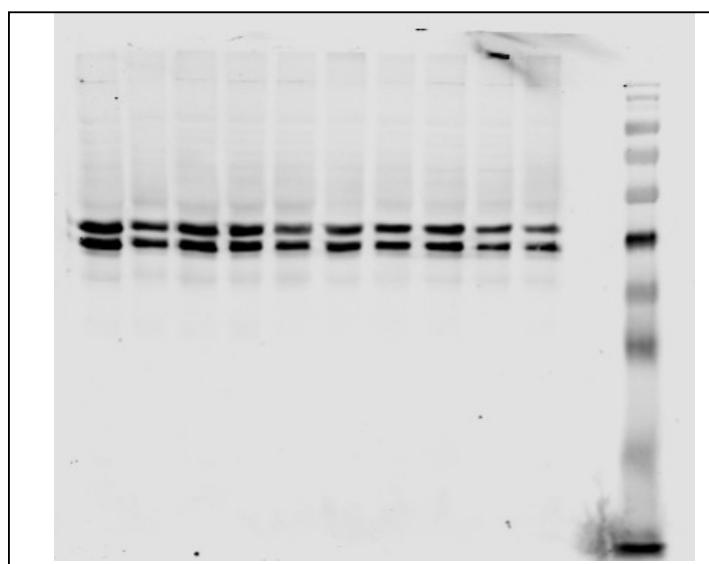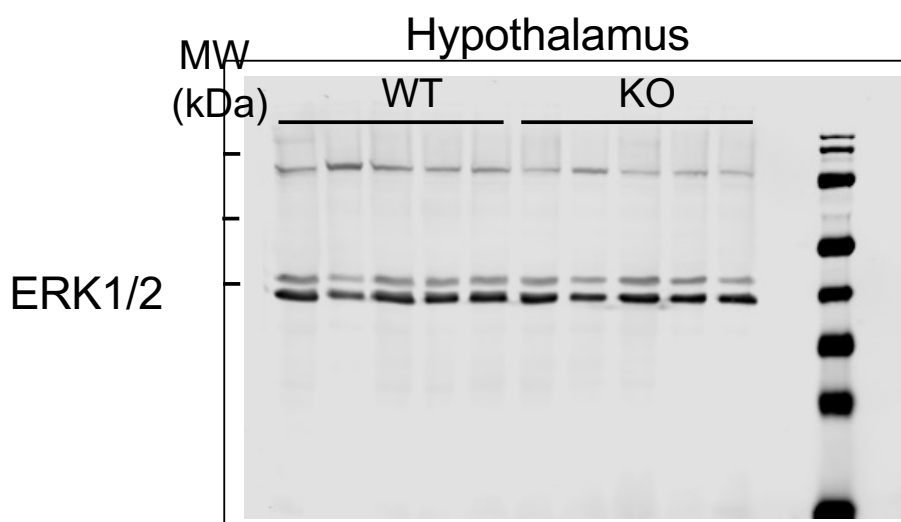

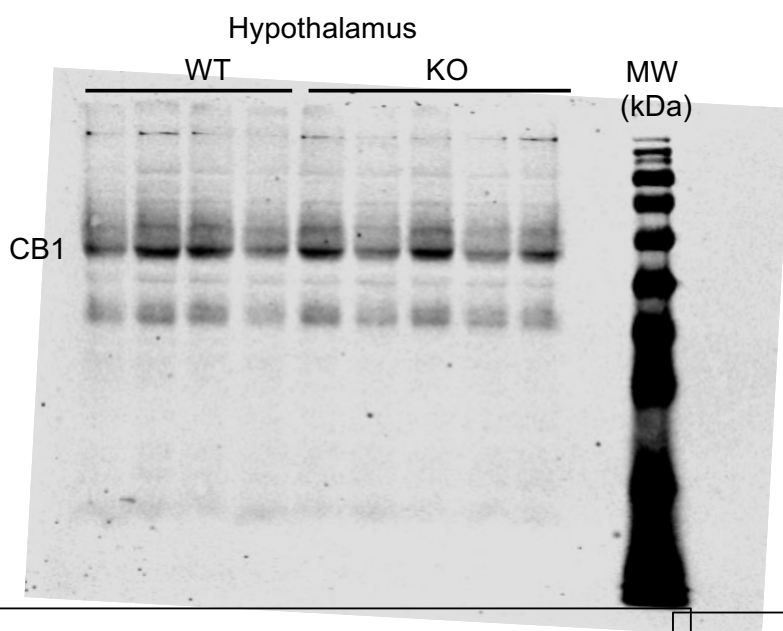

Fig. 5C

total

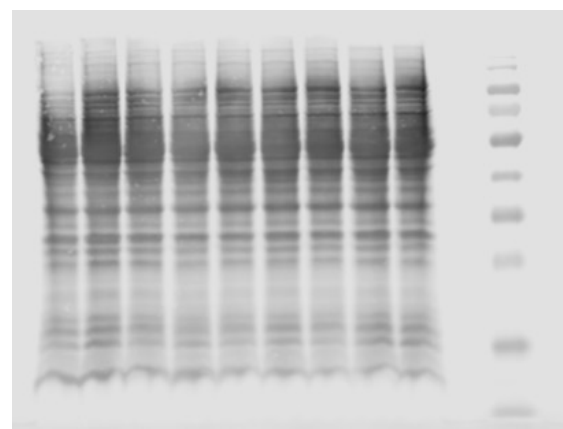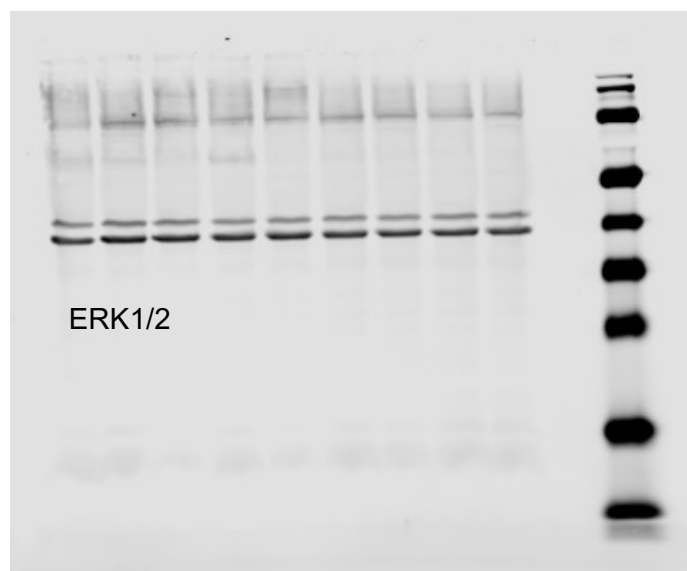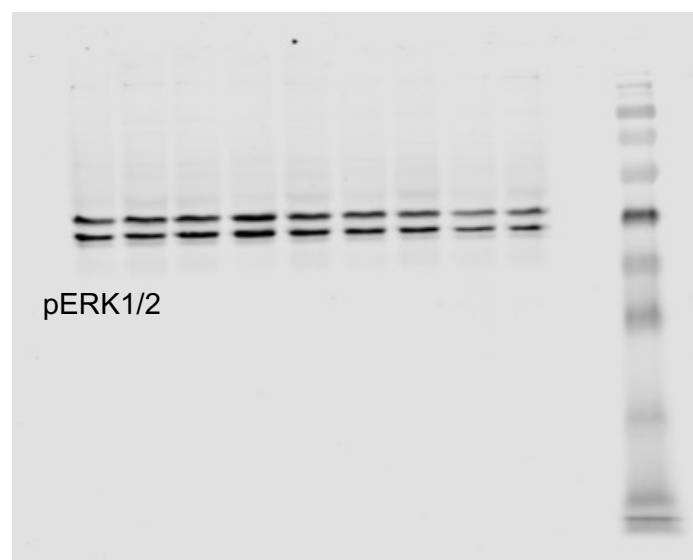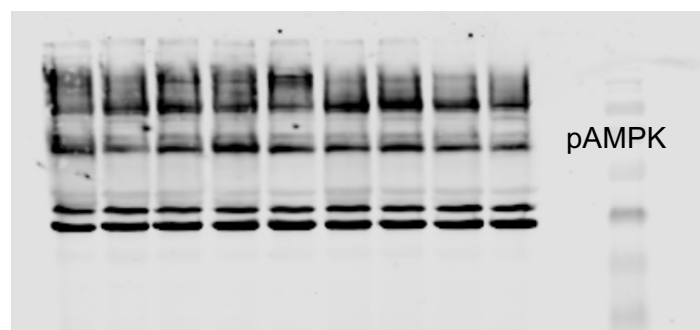

Supplement: Multimedia component 1 [file mmc1.pdf]
